# P300 with verbal and nonverbal stimuli in normal hearing adults

**DOI:** 10.1590/S1808-86942011000600002

**Published:** 2015-10-19

**Authors:** Camila Gonçalves Polo Massa, Camila Maia Rabelo, Carla Gentile Matas, Eliane Schochat, Alessandra Giannella Samelli

**Affiliations:** 1 Specialist in audiology. Clinical speech therapist; 2 Doctoral degree in science, Medical School, Sao Paulo University (FMUSP). Speech therapist, Physical Therapy, Speech Therapy, and Occupational Therapy Department, FMUSP; 3 Associate professor and faculty member, Physical Therapy, Speech Therapy, and Occupational Therapy Department, Medical School, Sao Paulo University (FMUSP); 4 Associate professor, Physical Therapy, Speech Therapy, and Occupational Therapy Department, Medical School, Sao Paulo University (FMUSP); 5 Doctoral degree in science, Medical School, Sao Paulo University (FMUSP). Assistant professor, Physical Therapy, Speech Therapy, and Occupational Therapy Department, FMUSP

**Keywords:** electrophysiology, event-related potentials, p300, speech perception

## Abstract

**Abstract:**

The P300 results from focusing attention on rare stimuli in the midst of other frequent stimuli; it tests recent attention and memory, both of which depend on discriminating among verbal or nonverbal stimuli.

**Aim:**

To compare the P300 with verbal and nonverbal stimuli in normal-hearing adults.

**Material and Method:**

A prospective study was made of 15 male subjects aged from 22 to 55, with no hearing complaints. The subjects underwent short and long latency (P300) auditory evoked potentials with verbal and non-verbal stimuli.

**Results:**

The mean P300 latency with verbal stimuli was significantly higher than the P300 with nonverbal stimuli. The P300 amplitudes were significantly lower for verbal compared with nonverbal stimuli.

**Conclusion:**

There were no differences between ears with respect to P300 latencies and amplitudes for both non-verbal and verbal stimuli. Latencies were higher with verbal stimuli; amplitudes had lower values.

## INTRODUCTION

Long latency auditory evoked potentials (LLAEP) are recorded electrical responses generated in the thalamus, auditory cortex, and cortical association areas; these structures are involved in discrimination, integration, and attention tasks. These potentials consist of a series of positive and negative peaks that occur at least 50 ms after a stimulus is initiated, and may be used as a clinical investigation tool to study neural mechanisms of auditory perception in subjects with normal and abnormal central nervous systems^1^, ^2^, ^3^, ^4^.

The P300 (cognitive potential) is the most frequently used LLAEP. It is a positive component of the potential, peaking at around 300 ms or more after a stimulus is initiated. Because it is an endogenous potential, the P300 affected by the functional use the brain makes of a sound stimulus and the attention level of a subject while it is being measured^5^,^6^. It is thought that the P300 is generated in structures of the frontal cortex, supratemporal auditory cortex, and the hypocampus^7^.

P300 is recorded by focusing attention on rare stimuli in the midst of other frequent stimuli. It is used to investigate attention and recent memory, both of which depend on discriminating between verbal or non-verbal stimuli^5^,^6^,^8^,^9^.

Cognitive potentials evoked by verbal stimuli may add information about the biological processes of speech processing, and are thus of major importance in clinical practice because extra information may be added to the standard behavioral evaluation – whether by cognitive, auditory, and/or linguistic reasons. Furthermore, evoked potentials by verbal stimuli help identify which specific speech signal aspects are not being coded, which may help guide rehabilitation and monitoring of patients^10^,^11^.

As the acoustic standards of verbal and non-verbal stimuli differ substantially, studies should compare how the central nervous system processes these stimuli, which can add to our understanding of abnormalities in the auditory system.

Thus, the purpose of this study was to compare the P300 evoked by verbal and non-verbal stimuli in normal hearing adult individuals.

## MATERIALS AND METHODS

The study abided by the standards of the Helsinki Declaration, and was approved by the institutional review board (no. 0479/09).

The study sample comprised 15 male subjects aged from 22 to 55 years (mean 33.4 years; standard deviation 12.47) that had no hearing complaints and generally good health, without a history of neurologic conditions; auditory thresholds were below or equal to 25 dBHL at 0.25 to 8 kHz.

Participants agreed to participate by signing a free informed consent form. The following procedures were carried out: meatoscopy, pure tone audiometry, immittance testing, and recording of short and long latency auditory evoked potentials.

Short and long latency auditory evoked potentials were measured by first cleaning the skin with abrasive paste, placing the electrodes with electrolytic paste and adhesive tape over the positions A1 (left mastoid), A2 (right mastoid), and Cz (vertex); the ground electrode was placed on the contralateral ear relative to the ear being tested. The electrode impedance value was equal to or lower than 5 kohms. The test equipment was an Auditory Evoked Potential (AEP) System, Navigator model, Bio-logic.

The brainstem auditory evoked potential (BAEP) was first done in all subjects to verify brainstem integrity; this was done to avoid distortions in the ensuing results, given that dysfunctions in the peripheral auditory system or brainstem may compromise P300 results. P300 was only measured after wave morphology and absolute wave I, III, and V latency values and interpeaks I-III, III-V, I-V values were shown to be within normal limits.

In BAEP testing, a click stimulus in rarefied polarity was presented through in-ear phones at 80 dBHL at a rate of 19 stimuli per second at 0.1 ms duration, totaling 2,000 stimuli. Testing was done twice in each ear for reproducibility of the tracings.

After BAEP testing, the P300 was tested in all subjects. Participants were asked to remain with their eyes closed (to avoid interference due to ocular movements) and to count the rare stimuli loudly (20% of all stimuli) that appeared randomly among frequent stimuli (80% of all stimuli) – the oddball paradigm – for each ear in turn.

Non-verbal stimuli were used (tone burst at 30 ms plateau and 10 ms rise/fall) at 1,000 Hz (frequent stimulus) and 2,000 Hz (rare stimulus). Verbal stimuli consisted of the syllables /ba/ (frequent stimulus) and /da/ (rare stimulus) at 75 dBHL, at a presentation speed of 11 stimuli per second. The analysis time was 800 ms, the high band pass filter was 1 Hz, the low pass band filter was 15 Hz, the gain was 50,000, and the sensitivity was 100 microvolts. For each stimulus type (verbal/ non-verbal) 300 stimuli were used. One recording was made in each side (ipsilateral) for each stimulus type; reproduction recordings were not made of these waves, as a second recording could cause subjects to become tired and compromise the overall results, which require attention.

The P300 is identified as a positive polarity wave with a post-stimulus latency of about 300 ms; it is obtained by subtracting the tracings of rare stimuli from those of frequent stimuli^5^. P300 latencies and amplitudes were analyzed for both stimulus types.

The statistical study consisted of using analysis of variance (ANOVA), which is a common parametric technique that compared the means through variance. Pearson's correction was also applied. The level of significance in this study was 0.05 (or 5%).

## RESULTS

### P300 with a non-verbal stimulus

Table 1 shows the results of P300 latencies and amplitudes after a non-verbal stimulus in the right and left ears.

**Table 1:** Mean, standard deviation (SD), and *p*-values of P300 latencies and amplitudes after non-verbal stimuli, for the right ear (RE) and left ear (LE).

Latency (ms)	RE LE
Mean	313.86 328.09
SD	33.33 22.20
*p*-value	0.232
Amplitude (μv)	RE LE
Mean	10.35 8.22
SD	3.29 3.15
*p*-value	0.119

These results show that there were no statistically significant differences between right and left ears in P300 latencies and amplitudes after non-verbal stimuli. Thus, right and left ears were groups together (Table 2).

**Table 2:** Mean and standard deviation (SD) of P300 latencies and amplitudes after non-verbal stimuli. Right and left ears are grouped together (n = 30).

Latency (ms)	Both ears
Mean	320.97
SD	28.63
Amplitude (μv)	Both ears
Mean	9.28
SD	3.33

### P300 with verbal stimuli

Table 3 shows the P300 latency and amplitude results after verbal stimuli in the right and left ears.

**Table 3:** Mean, standard deviation (SD) and p-values of P300 latencies and amplitudes after verbal stimuli in right ears (RE) and left ears (LE).

Latency (ms)	RE LE
Mean	356.02 341.88
SD	24.06 33.99
*p*-value	0.252
Amplitude (μv)	RE LE
Mean	5.86 7.36
SD	0.87 0.69
*p*-value	0.192

As in P300 results after non-verbal stimuli, P300 results after verbal stimuli were not statistically difference between right and left ears (Table 3). Thus, the ears were groups for subsequent analyses (Table 4)

**Table 4:** Mean and standard deviation (SD) of P300 latencies and amplitudes after verbal stimuli in the right and left ears grouped together (n = 30).

Latency (ms)	Both ears
Mean	348.95
SD	29.69
Amplitude (μv)	Both ears
Mean	6.61
SD	2.76

### Comparison of P300 after verbal and non-verbal stimuli

A comparison of data in Tables 2 and 4 – mean P300 latencies and amplitudes after verbal and nonverbal stimuli – yielded a *p*-value of 0.001. Thus, the mean P300 latency after verbal stimuli was significantly higher than P300 after non-verbal stimuli. Similarly, the amplitude *p*-value was 0.004, showing that the P300 wave amplitude was significantly lower after verbal stimuli.

### Correlation of P300 after verbal and non-verbal stimuli

Pearson's correlation coefficient was applied to check for any associations between P300 after verbal and non-verbal stimuli; the value was 0.099 (*p*-value 0.646), indicating a lack of association between both variables.

## DISCUSSION

At first, right and left ears were compared; there were no statistically significant latency and amplitude differences after verbal and non-verbal stimuli. These findings concur with those of Frizzo et al.^12^ who did not find statistically significant differences in the P300 of right and left ears after non-verbal stimuli in normal hearing adults.

Notwithstanding hemispheric differentiation and the unequal functional importance of brain hemispheres, there was no performance difference between right and left ears in this study. Kimura^13^ has stated that recognition of verbal auditory stimuli is a left hemisphere function, whereas non-verbal auditory stimuli are first processed in the right hemisphere.

Based on the assumption that stimuli presented to one ear are processed mainly by the contralateral ear, a verbal stimulus may result in left hemisphere predominance and superior auditory perception to the right. Non-verbal stimuli may result in right hemisphere predominance and superior auditory perception to the left.^14^ However, this difference is not noted in normal individuals; it may be seen in subjects with altered central auditory processing.^15^

We found no published paper comparing verbal stimulus P300 between ears. Few studies include verbal stimuli, and those that do did not mention ear or brain hemisphere differentiation.

Another finding was that P300 latencies by verbal stimuli were significantly higher than P300 latencies by non-verbal stimuli. Amplitudes by speech stimuli were significantly lower compared to non-verbal stimuli.

This was probably because discrimination of verbal stimuli (in this study, syllables /ba/ and /da/) is a more difficult and complex task compared to discrimination of non-verbal stimuli.^11^ Linden^6^ and Polich^9^ have stated that P300 latency increases when the discrimination targets are more difficult than the standard – latency is sensitive to the task processing demand. On the other hand, P300 amplitude is higher in easier tasks and decreases as tasks become more difficult^11^.

This scenario was not seen in Lew et al.'s^16^ 1999 study; in this case there was no statistically significant difference in a comparison of P300 latencies by verbal and non-verbal stimuli. This was probably because both tasks were relatively easy; the verbal rare stimulus for recording P300 was the word ‘mommy’ and the frequent stimulus was a 1,000 Hz tone.

Lew et al.^16^ found statistically significant higher P300 amplitudes with verbal stimuli compared to P300 with non-verbal stimuli. Our findings do not concur with these results; we found significantly higher P300 amplitudes with non-verbal stimuli.

The mean P300 latency values with non-verbal stimuli in the present study (320 ms) are close to the values suggested by McPherson,^7^ which are used at the audiology unit where this study was carried out. This author suggests a mean value of 315 ms for the age group in question.

We found no reference values in the literature for the P300 with verbal stimuli to compare with our findings. Thus, suggested values are based on the P300 wave latency with verbal stimuli for the study age group with 1 SD (319.26 ms – 378.64 ms) and 2 SD (289.57 ms – 408.33 ms). These are not normal reference values because the sample size was small in our study, but may be used for comparison purposes in future research.

Our findings also revealed lack of association among variables (non-verbal and verbal stimuli). This may be explained by acoustic differences between verbal and non-verbal stimuli, suggesting that these stimuli are processed differently in the nervous system^11^.

Because of the importance of the topic and the possible benefits of cognitive potentials with verbal stimuli, further studies are needed with larger samples and in different age groups to compare normal values with populations that present, among others, hearing, speech, and language disorders.

## CONCLUSION

Mean P300 latencies were higher after verbal stimuli compared to P300 latencies after non-verbal stimuli. On the other hand, mean amplitudes were lower after verbal stimuli compared to non-verbal stimuli. P300 latencies and amplitudes after non-verbal and verbal stimuli did not differ between ears.
